# Self-Emulsifying Drug Delivery Systems (SEDDS): Transition from Liquid to Solid—A Comprehensive Review of Formulation, Characterization, Applications, and Future Trends

**DOI:** 10.3390/pharmaceutics17010063

**Published:** 2025-01-05

**Authors:** Prateek Uttreja, Indrajeet Karnik, Ahmed Adel Ali Youssef, Nagarjuna Narala, Rasha M. Elkanayati, Srikanth Baisa, Nouf D. Alshammari, Srikanth Banda, Sateesh Kumar Vemula, Michael A. Repka

**Affiliations:** 1 Department of Pharmaceutics and Drug Delivery, School of Pharmacy, The University of Mississippi, Oxford, MS 38677, USA; puttreja@go.olemiss.edu (P.U.); rmelkana@go.olemiss.edu (R.M.E.);; 2 Department of Pharmaceutical Technology, Faculty of Pharmacy, Kafrelsheikh University, Kafrelsheikh 33516, Egypt; 3 Department of Pharmaceutics, College of Pharmacy, Northern Border University, Arar 91431, Saudi Arabia; 4 Department of Chemistry and Biochemistry, Florida International University, Miami, FL 33199, USA; 5 Department of Pharmaceutics, School of Pharmaceutical Sciences, Lovely Professional University, Phagwara 144411, Punjab, India; 6 Pii Center for Pharmaceutical Technology, The University of Mississippi, Oxford, MS 38677, USA

**Keywords:** self-emulsifying drug delivery systems, SEDDS, bioavailability enhancement, lipid-based drug delivery systems, SNEDDS, solid SEDDS, personalized medicine, 3D printing, in silico modeling, hot-melt extrusion

## Abstract

Self-emulsifying drug delivery systems (SEDDS) represent an innovative approach to improving the solubility and bioavailability of poorly water-soluble drugs, addressing significant challenges associated with oral drug delivery. This review highlights the advancements and applications of SEDDS, including their transition from liquid to solid forms, while addressing the formulation strategies, characterization techniques, and future prospects in pharmaceutical sciences. The review systematically analyzes existing studies on SEDDS, focusing on their classification into liquid and solid forms and their preparation methods, including spray drying, hot-melt extrusion, and adsorption onto carriers. Characterization techniques such as droplet size analysis, dissolution studies, and solid-state evaluations are detailed. Additionally, emerging trends, including 3D printing, hybrid systems, and supersaturable SEDDS (Su-SEDDS), are explored. Liquid SEDDS (L-SEDDS) enhance drug solubility and absorption by forming emulsions upon contact with gastrointestinal fluids. However, they suffer from stability and leakage issues. Transitioning to solid SEDDS (S-SEDDS) has resolved these limitations, offering enhanced stability, scalability, and patient compliance. Innovations such as personalized 3D-printed SEDDS, biologics delivery, and targeted systems demonstrate their potential for diverse therapeutic applications. Computational modeling and in silico approaches further accelerate formulation optimization. SEDDS have revolutionized drug delivery by improving bioavailability and enabling precise, patient-centric therapies. While challenges such as scalability and excipient toxicity persist, emerging technologies and multidisciplinary collaborations are paving the way for next-generation SEDDS. Their adaptability and potential for personalized medicine solidify their role as a cornerstone in modern pharmaceutical development.

## 1. Introduction

**Figure 1:**
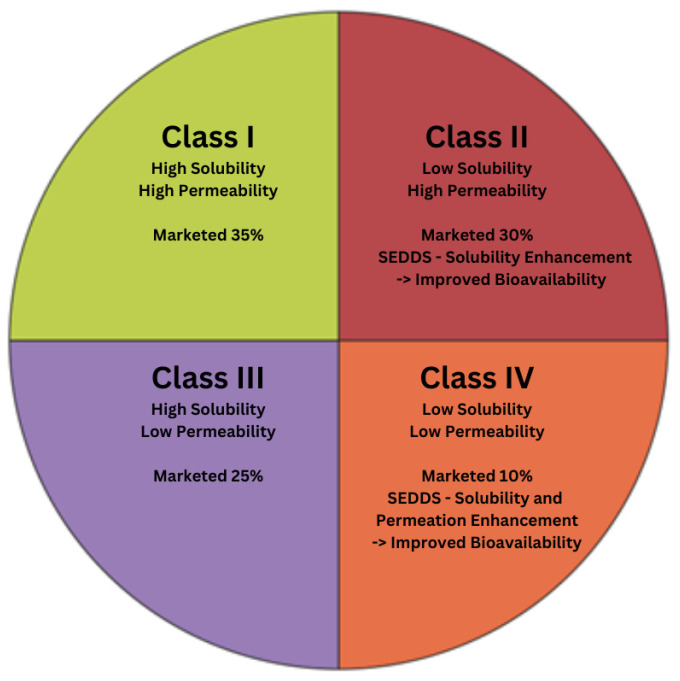
Biopharmaceutics classification system.

The oral route is the most favored route of administration due to ease of administration and thus improved patient compliance. However, low oral bioavailability due to poor aqueous solubility or permeability or both is still the significant challenge encountered by a formulator during developing a pharmaceutical product for the successful achievement of the desired in vivo performance for better therapeutic outcomes. The introduction of high-throughput screening to the drug discovery process has led to numerous lipophilic and poorly water-soluble new chemical entities. The poor solubility of these new chemical moieties poses a major challenge to formulation scientists during the formulation development of the brand and generic products. Generally, drug absorption is mainly dependent on two factors: solubility and permeability. In 1995, Amidon et al. introduced the Biopharmaceutics Classification System (BCS) that classified drugs into four categories based on these two factors as shown in Figure 1 [1,2]. Among the four BCS classes. Class II and IV drugs show poor aqueous solubility and low bioavailability. Thus, improving the dissolution profile of drugs within the BCS Class II and IV is a major challenge for researchers during the oral delivery of these drugs [3].

Numerous approaches are available for addressing these challenges, out of which, lipid-based drug delivery systems have been utilized as a principal formulation technique to deal with poor bioavailability issues [4]. The utilization of lipid-based formulations has been employed as a method to enhance the solubility and absorption of drugs administered orally, since it was shown that drugs with poor water solubility might be better absorbed when provided with lipid-rich foods [5]. Lipid-based formulations are seen as a viable strategy to improve the aqueous solubility and oral bioavailability of lipophilic drugs. The primary objective of these formulations is to keep drugs dissolved within the gastrointestinal tract [6]. Among the numerous lipid-based drug delivery systems, self-emulsifying drug delivery systems (SEDDS) are among the most extensively researched for oral administration.

Self-emulsifying drug delivery systems (SEDDS), characterized as emulsion concentrates, consist of a combination of drug, oils, surfactants, and occasionally co-surfactants [7]. While they do not qualify as emulsions in their original state, gentle agitation within the aqueous conditions of the stomach facilitates the formation of stable submicron-sized emulsions with ease. Consequently, SEDDS provide an opportunity for the solubilization of drugs that are poorly soluble in water. Their presence in the gastrointestinal tract, suspended in minute droplets, circumvents the dissolution phase of dispersed powder that restricts absorption [3]. In contrast to various lipid-based drug delivery systems, self-emulsifying drug delivery systems (SEDDS) exhibit a promising capability for an enhanced drug-loading capacity. This is attributed to the improved solubility of poorly soluble drugs characterized by an intermediate partition coefficient (2 < log P < 4) within the amphiphilic surfactants and co-surfactants components of the formulation [8]. Based on the preparation method, drug-loaded self-emulsifying drug delivery systems can be formulated as micro- (SMEDDS) or nano- (SNEDDS) formulations [9], which will be elaborated upon in the next section of the review.

Self-emulsifying drug delivery systems (SEDDS) represent a groundbreaking approach to addressing the ongoing issue of inadequate oral bioavailability in lipophilic drugs, especially those classified within BCS Class II and IV [10,11]. Through the formation of fine oil-in-water emulsions within the gastrointestinal tract, SEDDS effectively circumvent solubility and dissolution challenges, leading to enhanced drug solubilization and absorption. In clinical practice, this results in reliable therapeutic results, decreased variability in drug responses, and reduced side effects related to dosage [12]. SEDDS have shown remarkable adaptability in various therapeutic domains, facilitating the administration of drugs characterized by narrow therapeutic windows or significant lipophilicity. Furthermore, they offer versatility in formulation, supporting both rapid and controlled release characteristics while also improving patient adherence through oral administration. The significance of these characteristics illustrates the role of SEDDS in fulfilling unaddressed clinical demands, emphasizing their importance as a fundamental component in contemporary pharmaceutical research.

Conventional self-emulsifying drug delivery systems (SEDDS) are predominantly liquid formulations often contained within soft or hard gelatin capsules for oral use. All existing marketed formulations, including Sandimmune^®^ (cyclosporine), Norvir^®^ (ritonavir), and Fortovase^®^ (saquinavir), are based on liquid formulations [13,14]. A comprehensive list of marketed liquid SEDDS formulations is provided in Table 1, showcasing their clinical success in various therapeutic areas. While liquid SEDDS (L-SEDDS) offer several benefits, they encounter obstacles, including inadequate physical and chemical stability, potential leakage during storage, and restricted use in solid dosage forms, all of which can impact patient adherence and the feasibility of large-scale production [15]. To overcome these drawbacks, the transition to solid SEDDS has gained significant attention. Solid SEDDS (S-SEDDS), achieved via techniques like spray drying [16], melt extrusion [17], or adsorption onto solid carriers [18], combine the bioavailability advantages of liquid SEDDS with the stability, portability, and versatility of solid dosage forms. These systems offer higher drug-loading capacities, controlled release profiles, and compatibility with modern manufacturing processes, making them a robust alternative to conventional liquid SEDDS.

This review aims to provide a comprehensive analysis of self-emulsifying drug delivery systems (SEDDS), focusing on their evolution from liquid to solid forms, formulation strategies, characterization techniques, and diverse applications. By examining the fundamental differences between liquid and solid SEDDS, this review seeks to elucidate their respective advantages, challenges, and performance in enhancing bioavailability, controlling release, and enabling targeted drug delivery. The scope extends to addressing current challenges in SEDDS formulation, evaluating recent advancements, and exploring emerging trends and innovations that promise to shape the future of this drug delivery platform. Ultimately, this review intends to serve as a resource for researchers and formulation scientists, offering insights into the development and optimization of SEDDS for improved therapeutic outcomes.

## 2. Fundamentals of SEDDS

### 2.1. Composition and Mechanism

#### 2.1.1. Oils/Lipids

Oils are a vital component of SEDDS, as they not only have the tendency to strengthen the drug transport via intestinal lymphatic system to ultimately increase its absorption from the GI tract but also have the propensity to solubilize the lipophilic drug used in a SEDDS. Oils used in SEDDS can be classified into modified medium- and long- chain triglycerides of varying degrees of saturation or hydrolysis. These oils have higher drug-loading capacities, better emulsification characteristics when used in conjunction with an appropriate solubility-enhancing surfactant, and their degradation products are similar to the natural end products of intestinal digestion [12,19]. Natural, unmodified edible oils might seem to be a rational option for SEDDS development; however, these are inefficient in dissolving a high lipophilic drug load and possess inept self-emulsification properties [20].

Currently, lipidic systems comprising mono-, di-, and triglycerides, and their mixtures in varying proportions, with or without the fatty acid esters of propylene glycol, are being employed in the formulation of lipidic systems. Saturated and unsaturated fatty acids have been extensively used in their formulation. However, the SEDDS contain saturated fatty acids like caproic, caprylic, capric, lauric, and myristic acid. These novel semi-synthetic lipidic constituents are amphiphilic and offer additional surfactant-like properties [21]. The lipidic constituents in SEDDS are mostly the mono-, di-, or triglyceryl derivatives with HLB values ranging between 1 and 6 and melting points varying between −78 °C and +78 °C. In addition, SEDDS can also be fabricated using the mixtures of mono-, di-, and triglycerides with fatty acid esters of polyethylene glycol (PEG) having HLB values between 3 and 18 [22].

Previous studies have established long-chain lipids were found to resist drug precipitation when compared to medium-chain lipids [23]. Mixed micelles are formed when the lipidic components trigger the secretion of bile and pancreatic juices. These mixed micelles, comprising bile salts, phospholipids, and cholesterol, enhance the solubility of poorly soluble components [9]. Thus, oils not only serve as carriers for lipophilic drugs but also enhance solubility, promote lymphatic transport, and significantly improve drug absorption, making them an indispensable component of SEDDS.

#### 2.1.2. Surfactants

Surfactants play a crucial role in SEDDS by reducing the interfacial tension between the oil and water phases, thereby creating a stable emulsion. Thus, these substances act as emulsifiers and help in the regulation of the droplet size and thus the release rate and stability of the system [24]. Moreover, surfactants also inhibit the CYP3A4 biotransformation behaviors via extracellular membrane signaling [25]. Additionally, non-ionic surfactants can also act as P-glycoprotein efflux inhibitors by inhibiting the ATP-binding cassette. Furthermore, non-ionic surfactants enhance drug absorption by disrupting the lateral membrane packing of the gastrointestinal lumen and enabling passive transport across the gut wall [26].

Surfactants for SEDDS are chosen based on the safety and HLB profiles of the components. A high HLB value of a surfactant corresponds to a higher aqueous affinity, which is vital for presenting a lipidic drug delivery system with enhanced self-emulsifying properties. Such a surfactant helps in quicker o/w droplet development and swift dispersal of the formulation in the aqueous media for the immediate formation of o/w droplets [27]. Thus, precipitation of the drug in the gut environment can be avoided and efficient absorption can be achieved by holding the drug at the site of absorption for an extended period of time [28]. Non-ionic surfactants are regarded as safer than ionic surfactants while also stabilizing systems formed over a broad range of pH and ionic strengths [29,30].

Non-ionic, amphiphilic surfactants are preferred for the formulation of S-SEDDS. The amphiphilic nature of the surfactant aids in dissolving an increased load of the poorly water-soluble active substance in the gastrointestinal lumen while avoiding precipitation [12]. Some of the most used surfactants include Gelucire^®^ 50/13 (lauroyl macrogol-32-glyceride), Gelucire^®^ 44/14, Gelucire^®^ 48/16, Cremophor^®^ EL (polyoxy-35-castor oil), Cremophor^®^ RH 40 (polyoxyethylene hydrogenated castor oil), Labrasol^®^ (caprylocaproyl macrogol-8-glyceride), Tween 80 (polyoxyethylene (20) sorbitan monooleate), and Pluronic F127 (poloxamer 407).

For SEDDS, the effective concentration of the surfactant should range between 30 and 60%, as a higher concentration might irritate the gastric mucosa and cause cytotoxicity. Nevertheless, an elevated concentration of surfactants might be needed for effective dissolution of the higher concentrations of hydrophobic drugs to develop an effective SEDDS. However, a mixture of surfactants used to prepare SEDDS improves the solubilization efficiency of the compound and can thus reduce the total quantity of the surfactants required to develop an efficacious self-emulsifying system [24,29]. Therefore, surfactants not only enhance emulsification by reducing interfacial tension but also stabilize the formulation, improve drug absorption through inhibition of the efflux mechanisms, and enable the rapid formation of fine oil-in-water droplets in the GI tract.

#### 2.1.3. Co-Surfactants/Co-Solvents

SEDDS contain a large amount of hydrophilic surfactants to enable the dissolution of the hydrophobic drug substance. To support the dissolution of these hydrophilic surfactants and the drug substance in lipids, co-surfactants like alcohols of medium to short chain lengths (C3–C8; PEG, propylene glycol and polyethylene glycol) are used, as they are appropriate for oral drug delivery. Co-surfactants act by improving the emulsification process by enhancing the fluidity between the emulsion phases, thereby preventing liquid crystallization [31]. The co-surfactant is selected depending on its capability to maximize the emulsification area coupled with the chosen surfactant. The surfactant and co-surfactant are adsorbed at the interface preferentially, thereby providing a mechanical barrier to coalescence, as well as reducing the interfacial energy. Thus, the thermodynamic stability of the emulsion increases as the energy of the whole system drops down [32]. Co-surfactants also help in reducing the gastrointestinal distress and improve the penetrability of the dispersion media [13]. Higher concentrations of surfactants and/or co-surfactants in comparison to oils leads to the formation of SMEDDS [33,34]. Earlier findings have also pointed to the failure of the formation of SNEDDS in the absence of co-surfactants [35].

The selection of surfactants and co-surfactants for an effective SEDDS should be based on the emulsification efficiency and not the low solubilizing capacity for the hydrophobic drug [32,36]. Thus, co-surfactants synergize with surfactants to improve emulsification, increase thermodynamic stability, and reduce drug precipitation, ensuring consistent drug delivery and absorption.

#### 2.1.4. Drugs

Typically, drugs categorized under BCS Class II and Class IV are considered for the formulation of SEDDS. Nonetheless, SEDDS may not be appropriate for every drug, and addressing the issue of drug sedimentation within the gastrointestinal tract is also a key consideration in the design of SEDDS [37]. According to the established guidelines, the log P of drugs in SEDDS formulations should ideally exceed 5 to ensure favorable solubility in the lipid, surfactant, and co-surfactant phases [38]. Compounds with a log P greater than 2 generally exhibit sufficient solubility in these components, enhancing their potential for absorption [39]. In summary, drugs with a log P greater than 5 are more likely to demonstrate enhanced solubility and absorption in self-emulsifying drug delivery systems. Once combined with SEDDS, these drugs can be efficiently absorbed by intestinal epithelial cells and subsequently integrated into chylomicrons. Therefore, the selection of appropriate drugs for SEDDS, based on their physicochemical properties, is critical to achieving enhanced solubility, improved absorption, and effective therapeutic outcomes in self-emulsifying systems. Table 2 represents the components, including oils, surfactants, co-surfactants, and drugs, commonly used in recently investigated SEDDS formulations.

#### 2.1.5. Mechanism of Drug Absorption and Bioavailability Enhancement by SEDDS

Upon administration, SEDDS initially disintegrate and subsequently self-emulsify with mild agitation in the stomach. Lipids and various lipophilic excipients influence absorption and, consequently, the bioavailability of lipophilic drugs administered via the oral route through multiple mechanisms. The components of SEDDS interact with the enterocyte-based transport system, facilitate lymphatic transport of the drug, and alter the intestinal composition and environment [48]. Dispersion in the gastrointestinal tract (GI), lipid digestion, and the solubility of the drug and lipidic components all have a role in the absorption of drugs via SEDDS.

Drug solubility in the small intestine is mostly influenced by lipid-digesting behavior. The digestion of lipids is completed by pancreatic lipase in the small intestine, after gastric lipase in the stomach partially breaks them down. Bile salts and cholesterol are secreted in the duodenum in response to lipids and lipid digestion products, facilitating the micellar solubilization of hydrophobic drugs. Lipid digestion products, such as monoglycerides and diglycerides, are internalized within colloidal structures, including mixed micelles, micelles, and vesicles, which subsequently leads to the solubilization of the drug [49,50].

Moreover, drug absorption occurs through the apical membrane of enterocytes via numerous mechanisms. The intestinal lumen and the enterocyte apical membrane are separated by an unstirred water layer (UWL), which acts as a significant barrier to lipophilic drug transport. Micellar solubilization, on the other hand, makes free fatty acids, monoglycerides, and drugs more soluble in water. This makes it much easier for these substances to move across the UWL in large amounts. Only passive diffusion or carrier-mediated transport transfers free drugs and fatty acids across the apical membrane of enterocytes, not micellar absorption. Micelles are absorbed intact through vesicular-mediated transport, which is when micelles connect with transport proteins on the top membrane of enterocytes [9,51].

**Figure 2:**
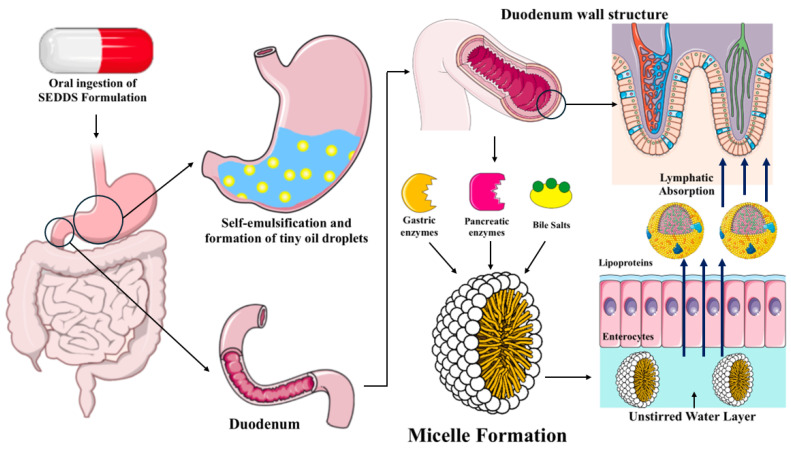
Mechanisms of drug absorption and bioavailability enhancement by SEDDS.

Triglycerides are formed when monoglycerides and free fatty acids enter the enterocytes. The triglycerides are converted into lipoproteins (LPs) and then released into the lamina propria through exocytosis from enterocytes. It is easy for drug molecules with a log P > 5 to bind to the LPs and be transported along with them. The tight endothelial junctions and basement membrane of blood capillaries preclude the efficient uptake of lipoproteins into the vascular system. Nevertheless, they can readily experience lymphatic absorption in areas with extremely permeable large inter-endothelial junctions. Drugs with high lipophilicity (log P > 5) circumvent entrance through the portal vein into systemic circulation and preferentially utilize lymphatic pathways coming from the intestine. Intestinal lymph carries drugs straight into the bloodstream, preventing them from entering the liver. Consequently, modifying the transport route for absorption also inhibits first-pass metabolism [51,52]. Hence, the mechanisms clearly show that SEDDS is a very effective approach for enhancing the bioavailability of lipophilic drugs and avoiding their first-pass metabolism. By leveraging these mechanisms—self-emulsification, micellar solubilization, lipid digestion, and lymphatic transport—SEDDS effectively enhance the bioavailability of poorly soluble drugs while avoiding first-pass metabolism. Figure 2 visually illustrates these processes, highlighting the transformation of SEDDS into fine oil droplets and their subsequent absorption pathways in the gastrointestinal tract.

### 2.2. Classification of SEDDS

#### 2.2.1. Conventional SEDDS, SMEDDS, and SNEDDS

Self-emulsifying drug delivery systems (SEDDS) are categorized based on their droplet size, stability, and bioavailability. These systems also differ significantly in the types of oils, surfactants, and co-surfactants used, which influence their self-emulsification efficiency and overall performance. Conventional SEDDS primarily use long-chain triglycerides, while SMEDDS and SNEDDS incorporate medium- and short-chain triglycerides, enabling improved emulsification and absorption. Furthermore, SMEDDS and SNEDDS rely on high-HLB surfactants and specific co-surfactants to achieve superior stability and bioavailability [30]. Table 3 provides a detailed comparison of these systems, including their key formulation components and characteristics.

#### 2.2.2. Lipid Formulation Classification System (LFCS)

Lipid-based formulations are systematically classified under the Lipid Formulation Classification System (LFCS) to facilitate their selection and optimization based on drug characteristics and therapeutic goals. The LFCS categorizes formulations into four types (I–IV), with SEDDS primarily falling under Types II and III. Conventional SEDDS correspond to Type II formulations, while SMEDDS and SNEDDS are classified under Type IIIa and IIIb, respectively, based on their surfactant content and emulsification efficiency [2,30]. Table 4 provides a detailed comparison of LFCS types, their composition, behavior in aqueous media, and their corresponding SEDDS categories.

In Type I formulations, the composition consists solely of oil and drugs. This formulation lacks surfactants, which will inhibit emulsification in biological systems. As a result, there will be reduced drug dissolution within mixed micelles and an increase in drug precipitation in the gastrointestinal tract post-digestion. Furthermore, pancreatic lipase functions as an interface enzyme [55,56]. However, in the gastrointestinal tract, only bile salts and phospholipids are involved in the emulsification process for Type I preparations. This involvement may impede the interaction between pancreatic lipase and emulsion, resulting in a slower and less effective lipolysis [56,57]. Consequently, effective digestion is essential to guarantee the bioavailability of drugs in Type I formulations.

The formulation of Type II consists of an oil phase combined with a water-insoluble surfactant. It is noteworthy that lipophilic surfactants with a HLB < 12 can enhance the dissolution of drugs within self-emulsifying drug delivery systems, as well as facilitate the emulsification and dispersion of these systems in aqueous environments. However, these surfactants are not sufficiently hydrophilic, leading to inadequate solubilization of the drugs in vivo. This results in drug precipitation within the body and subsequently low oral bioavailability.

Type III formulations incorporate oil, co-surfactant, and surfactant with a hydrophilic–lipophilic balance exceeding 12. It has the potential to create a stable O/W emulsion with minimal agitation in vivo. Consequently, Type III formulations can readily transition into the aqueous phase following dispersion and steatolysis, demonstrating superior potential for oral absorption in comparison to Type I and Type II formulations [8].

Type IV preparation consists of either a pure surfactant or a combination of surfactant and co-surfactant. This formulation exhibits excellent dissolution properties for pharmaceuticals. Nevertheless, the preparation of Type IV exhibits instability in the gastrointestinal tract when subjected to dilution. Consequently, the drugs in this formulation would lead to drug precipitation, which would not enhance the pharmacokinetics [57]. Elevated levels of the surfactant in Type IV formulations could lead to gastrointestinal toxicity [57]. Fortunately, the use of a co-surfactant aids in minimizing the amount of surfactants required.

## 3. Formulation Strategies

### 3.1. Liquid SEDDS (L-SEDDS)

Liquid SEDDS are widely used for enhancing the oral bioavailability of poorly water-soluble drugs. Their formulation involves simple mixing of oils, surfactants, and co-surfactants, often accompanied by a homogenization step to ensure a uniform and stable mixture. This is typically achieved by dissolving the drug in the selected components with mild heating (30–50 °C) to aid solubilization, followed by thorough blending using high-shear mixing or magnetic stirring [21,58]. This straightforward preparation process makes liquid SEDDS easier to formulate compared to more complex drug delivery systems. These formulations improve drug solubilization in gastrointestinal fluids, bypass the dissolution phase, and provide faster absorption. Additionally, liquid SEDDS enable the delivery of drugs with a narrow therapeutic window, ensuring more consistent plasma concentrations and reduced inter- and intra-patient variability. The ability to incorporate lipophilic drugs with high log P values also makes liquid SEDDS an attractive option for a wide range of therapeutic agents [59].

The selection of excipients is a critical prerequisite for formulating SEDDS, as only specific combinations of these excipients lead to efficient self-emulsifying systems. Preliminary studies involve determining the solubility of the drug in various oils and surfactants. A series of SEDDS formulations were prepared with different oil and surfactant combinations, and their in vitro self-emulsification properties are studied by adding them to water under mild agitation. Droplet size analysis is then conducted to assess the quality of the formulations. A pseudo-ternary phase diagram is constructed to identify the most efficient self-emulsification region, from which an optimized formulation is selected. This optimized formulation is further analyzed for bioavailability, comparing it with a reference formulation to evaluate the improvement in oral absorption [7,17]. The efficiency of the oral absorption depends on several formulation-related parameters, such as surfactant concentration, oil-to-surfactant ratio, emulsion polarity, droplet size, and charge, which collectively determine the self-emulsification ability of the system [60]. The optimized formulation is then prepared using the most suitable method, ensuring consistency and scalability for enhanced bioavailability and therapeutic efficacy.

Despite their benefits, liquid SEDDS suffer from several limitations. Stability issues such as phase separation, drug precipitation upon storage, and chemical degradation are common challenges. The liquid nature of these formulations also requires encapsulation in soft or hard gelatin capsules, which may leak during storage, compromising the formulation’s integrity. Additionally, these systems are unsuitable for moisture-sensitive drugs, and their reliance on high surfactant concentrations can cause gastric irritation. Storage conditions, including temperature and humidity, further complicate the shelf-life and scalability of liquid SEDDS [61]. These limitations, coupled with the requirement for improved stability and patient compliance, highlight the growing need for solid SEDDS. Transforming liquid SEDDS into solid forms offers solutions to these challenges by combining the bioavailability benefits of liquid formulations with the stability and convenience of solid dosage forms [61]. Table 5 provides a detailed comparison of the key aspects of liquid and solid SEDDS, highlighting their unique advantages and limitations.

### 3.2. Solid SEDDS (S-SEDDS)

Transforming liquid SEDDS into solid forms provides several advantages, including enhanced drug solubilization, improved safety, controlled release, and commercial benefits. Solidified SEDDS are more stable, easier to handle, and better suited for industrial scaling. One key benefit is better drug solubilization and dissolution. Liquid SEDDS often suffer from drug crystallization and precipitation in the gastrointestinal (GI) tract, leading to inconsistent pharmacokinetics [66]. To address this, supersaturated SEDDS (super-SEDDS) have been developed, which allow higher drug loading by combining liquid lipids with solid carriers like porous colloids to stabilize the drug and prevent crystallization [67].

Solidifying SEDDS also enables controlled drug release, regulating the drug release through mechanisms like diffusion from lipid droplets or erosion from solid carriers. This improves bioavailability, as seen in formulations like spray-dried SEDDS with PLGA nanoparticles, which provide dual-phase release and better absorption than liquid SEDDS [68]. Safety is another benefit, as solid SEDDS reduce the need for the high surfactant levels required in liquid SEDDS, which can cause toxicity with long-term use. By using solid stabilizers, toxicity risks are lowered while maintaining drug solubility. Additionally, solid SEDDS offer improved oxidative stability, protecting lipids from degradation caused by exposure to air or moisture, preserving their integrity. From a manufacturing standpoint, solid SEDDS simplify packaging, administration, and scaling. They can be produced as sachets, capsules, or tablets using standard equipment, improving patient compliance, especially for pediatric use, and reducing production costs. Solidifying SEDDS thus offers significant benefits in terms of stability, manufacturing, and ease of use [54]. Table 6 highlights examples of solid self-emulsifying drug delivery systems (S-SEDDS) formulations, showcasing the use of various active pharmaceutical ingredients (APIs), solid carriers, and preparation methods.

#### 3.2.1. Solid Carriers

The solid carrier in a S-SEDDS system helps in holding on to the liquid components of the system. The solid carriers act by encapsulating the scattered lipids before the drying phase or by absorbing the liquid components. The ideal characteristics of a solid carrier include the superior ability to hold onto the liquid components, optimum flow properties, and sufficient mechanical strength. The porosity, hydrophilicity, and surface area of the solid carrier also play a role in the selection of the solid carrier. Moreover, the redispersion properties of the solid carrier modulate the release of the drug from the system and the biopharmaceutical performance. Solid carriers can be subdivided into water-soluble and water-insoluble carriers. Polymers, polysaccharides, and protein-based carriers constitute water-soluble carriers, while water-insoluble solid carriers include porous and non-porous silica adsorbents and some aluminosilicates and carbonates [69].

**Table 6:** Examples of solid SEDDS (S-SEDDS) formulations.

API	Solid Carrier	Method of Preparation	Reference
Dexibuprofen	Silicon Dioxide (Aerosil^®^ 200)	Spray Drying	[70]
Lysozyme	Magnesium Aluminometasilicate (Neusilin^®^ UFL2)	Adsorption using Mortar-Pestle	[71]
Flubiprofen	Silicon dioxide (Aerosil^®^ 200); Magnesium stearate; Polyvinyl alcohol (PVA); Sodium carboxymethyl cellulose (Na-CMC); Hydroxypropyl-β-cyclodextrin (HP-β-CD)	Spray Drying	[72]
Cannabidiol	PEO N80 (Polyethylene oxide), and Soluplus^®^ (polyvinyl caprolactam–polyvinyl acetate–polyethylene glycol graft copolymer)	Hot-melt Extrusion	[73]
Curcumin	Soluplus^®^; Magnesium Aluminometasilicate (Neusilin^®^ UFL2)	Spray Drying	[74]
Carbamazepine	Diatom silica	Adsorption using Mortar-Pestle	[75]
Glimepiride	Silicon dioxide (Aerosil^®^ 200)	Spray Drying	[76]
Docetaxel	Lactose	Spray Drying	[77]
Quetiapine Fumarate	Soluplus^®^ and Hydroxypropyl Cellulose (Klucel™ EF)	Hot-melt Extrusion	[17]
Chlorthalidone	Silicon dioxide (Aerosil^®^ 200)	Spray Drying	[78]
Quercetin	Silicon dioxide (Aerosil^®^ 300)	Spray Drying	[79]
Carvedilol	Silicon dioxide (Aerosil^®^ 200); Hydroxypropyl Methylcellulose Acetate Succinate, HPMCAS (AquaSolve AS^®^ LG); Hydroxypropyl Cellulose (Klucel™ EF); Microcrystalline cellulose pH 101; Talc	Hot-melt Extrusion	[80]
Meloxicam	Mannitol; Fumed Silica	Lyophilisation	[81]
Orlistat	Silicon dioxide (Aerosil^®^ 200); Microcrystalline cellulose PH 102 (Avicel PH 102)	Lyophilisation	[82]
Flubiprofen	Silicon dioxide (Aerosil^®^ 200); Dextran	Spray Drying	[83]
Indomethacin	Silicon dioxide (Syloid^®^ XDP 3150); Magnesium Aluminometasilicate (Neusilin^®^ UFL2); Synthetic Calcium Silicate (Florite^®^ PS-200)	Hot-melt Extrusion	[84]
Azithromycin	Lactose; Mannitol; Calcium Carbonate; Silicon dioxide (Aerosil^®^ 200)	Adsorption using Mortar-Pestle	[62]
Resveratrol	Silicon dioxide (Aerosil^®^ 200)	Hot-melt Extrusion	[85]
Paclitaxel	Silicon dioxide (Aerosil^®^ 200); Dextran	Spray Drying	[86]
Atorvastatin	Lactose (Lactochem^®^ powder)	Spray Drying	[87]
Furosemide	Microcrystalline Cellulose	Adsorption using Mortar-Pestle	[88]
Clozapine	Silicon dioxide (Aerosil^®^ 200); Microcrystalline Cellulose	Adsorption using Mortar-Pestle	[89]
Ibuprofen	Magnesium Aluminometasilicate (Neusilin^®^ US2); Starch 1500^®^; Microcrystalline Cellulose (Avicel PH^®^ 102)	Hot-melt Extrusion	[43]
Fenofibrate	Magnesium Aluminometasilicate (Neusilin^®^ US2)	Hot-melt Extrusion	[15]
Celecoxib	Calcium Silicate; Silicon dioxide (Aerosil^®^ 200)	Spray drying; Fluid Bed Granulator	[90]

#### 3.2.2. Typical Formulation Methods of Solid SEDDS (S-SEDDS)

##### Hot-Melt Extrusion

One novel manufacturing process is hot-melt extrusion (HME), in which a material is melted or softened before being forced through an aperture at a high pressure and temperature to create a solid, uniformly shaped product [91]. The physical properties are altered as the material is driven through an aperture on a hot-melt extruder under temperature and pressure control. An extrusion barrel, a revolving screw, a motor, a heater, and an aperture are the components that make up the extruder [92,93]. Polymers melt due to the friction and heat generated by the screws in the barrel. The following is the procedure for preparing S-SEDDS using HME—(a) liquid SEDDS adsorption onto a meltable binder (e.g., HPC and MCC), (b) with the application of heat and pressure, the solid substance will undergo melting and softening, and (c) transformation of the molten material into a semisolid mass, followed by extrusion into granules or pellets [94]. Extruders can either have one screw or two screws firmly affixed to them. There are two distinct ways to feed: flood feeding, in which the feeder is placed above the feed throat, and starve feeding, in which the mass flow rate of the feed system is used. Typically, a starving feed is used for a twin-screw extruder, whereas a flood feed is used for a single-screw extruder. Both counter-rotating (screws rotate in opposite directions) and co-rotating (screws rotate in the same direction) configurations are possible in a twin-screw extruder. The die is the last component of the extruder and is used to shape the molten material as it exits the extruder. The die shapes and sizes extrudate into the desired form, such as a film, thread, granule, or sheet. HME has many benefits, such as its high drug loading, improved content uniformity, a solvent-free method, high efficiency, and short production time, and it provides opportunity to prepare novel formulations. Nevertheless, significant energy input can degrade APIs, rendering the process inappropriate for thermolabile drugs [95].

##### Lyophilization

Lyophilization is another term for “freeze drying”. This method is employed in the pharmaceutical industry to improve the stability of drug formulations. This method of drying relies on the solvent being frozen and then sublimated to remove moisture. S-SEDDS are produced by freezing the liquid self-emulsifying formulation and then sublimating the frozen aqueous state at low temperatures and pressures [96]. Lyophilization involves the use of cryoprotectants, such as mannitol, dextrose, and lactose, as solid carriers to enhance the stability of the end product [97]. The lyophilized self-emulsifying drug delivery systems (SEDDS) exhibit favourable stability and flow properties. This procedure exhibits a significantly elevated level of desiccation efficacy, with the capability to remove 95–99.5% of the water content. Additionally, it is well suited for thermolabile pharmaceutical compounds [98].

##### Spray Drying

The process of spray drying is a widely recognized commercial method utilized in the production of solid pharmaceutical powders. The spray drying process involves the transformation of a liquid feed solution into solid powders through the controlled application of temperature and airflow within a drying chamber. Spray drying can be used to solidify liquid SEDDS by adding certain solid carriers in addition to the liquid SEDDS fundamental components. The benefits of spray drying include single-step processing, low cost, production of uniformly sized particles, and improved dissolving profiles. Spray drying is limited in its applicability for thermally sensitive drugs and proteins, which may undergo degradation during the process. Additionally, lower yields may be obtained in certain cases [99].

##### Adsorption onto Solid Carriers

Adsorption is a thermodynamically favorable phenomenon that involves the accumulation of adsorbate molecules onto the surface of an adsorbent material. This process is characterized by a release of heat energy and is highly dependent on the surface properties of the adsorbent material. The adsorption technique is considered the most cost-effective and uncomplicated approach for developing solid self-microemulsifying drug delivery systems (SMEDDS). This method produces solid SMEDDS that are stable and free flowing. The adsorbent typically employed exhibits a porous morphology and possesses a substantial liquid adsorption capability. Among the frequently utilized adsorbents are Neusilin US2 (magnesium aluminometasilicate), Aerosil 200 (silicon dioxide), Florite RE (porous silicate carrier), Syloid 244 FP (porous silicon dioxide), colloidal silica, dextran, etc. Neusilin US2 has been extensively studied as an adsorbent owing to its exceptional flow characteristics, high liquid adsorption capacity, and compaction properties. Hard gelatin capsules may be filled with solid SMEDDS, or they can be combined with other excipients and compacted into tablets [100].

##### Three-Dimensional Printing

The integration of 3D printing technology has emerged as a revolutionary approach for the formulation of solid SEDDS, enabling precise and customizable drug delivery systems [101,102]. Techniques such as fused deposition modeling (FDM) and semisolid extrusion-based 3D printing allow the conversion of liquid SEDDS into solid dosage forms, facilitating enhanced stability and patient compliance [103]. For instance, 3D-printed hollow tablets containing self-nanoemulsifying formulations have been developed to deliver poorly soluble drugs like curcumin with controlled release profiles [104]. By optimizing variables such as wall thickness, nozzle size, and printing parameters, these systems can achieve tailored drug release, improved bioavailability, and protection against degradation in gastrointestinal conditions. The ability to combine lipid-based carriers with 3D-printed matrices provides an innovative platform for creating personalized, multi-drug delivery systems, addressing the limitations of conventional methods [105,106].

**Figure 3:**
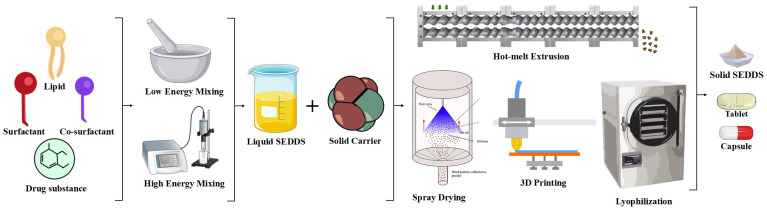
Transformation of liquid SEDDS to solid SEDDS using various techniques.

Figure 3 visually illustrates the transformation of liquid SEDDS into solid SEDDS through various innovative techniques. The diagram highlights key processes such as low- and high-energy mixing for the preparation of liquid SEDDS, followed by solidification methods like hot-melt extrusion, spray drying, adsorption onto solid carriers, lyophilization, and 3D printing. These processes enable the development of solid dosage forms, including capsules and tablets, enhancing the stability, portability, and application range of SEDDS formulations. This comprehensive representation emphasizes the versatility and potential of S-SEDDS in addressing contemporary pharmaceutical challenges.

## 4. Characterization Methods for SEDDS

Comprehensive characterization is essential for ensuring the quality, stability, and bioavailability enhancement potential of SEDDS formulations. Various techniques are employed to evaluate the physical and functional attributes of both liquid and solid SEDDS. The characterization techniques employed for liquid SEDDS are equally applicable to solid SEDDS, ensuring consistency in evaluating their performance. Upon reconstitution or dispersion in aqueous media, solid SEDDS can be assessed using a range of physicochemical methods. Key tests include self-emulsification efficiency, which examines the ability of the formulation to form an emulsion upon contact with gastrointestinal fluids, and droplet size analysis, which provides insight into the emulsification quality and potential for drug absorption. Cloud point measurement helps determine the stability of the formulation at varying temperatures, while in vitro drug release studies assess the dissolution and release profiles of the active pharmaceutical ingredient (API) [107]. These evaluations ensure that SEDDS retain the desirable properties, such as rapid emulsification, small droplet sizes for enhanced bioavailability, and stable drug release characteristics. By applying these comprehensive characterization methods, formulation scientists can optimize SEDDS for improved therapeutic efficacy and patient compliance [53].

**Figure 4:**
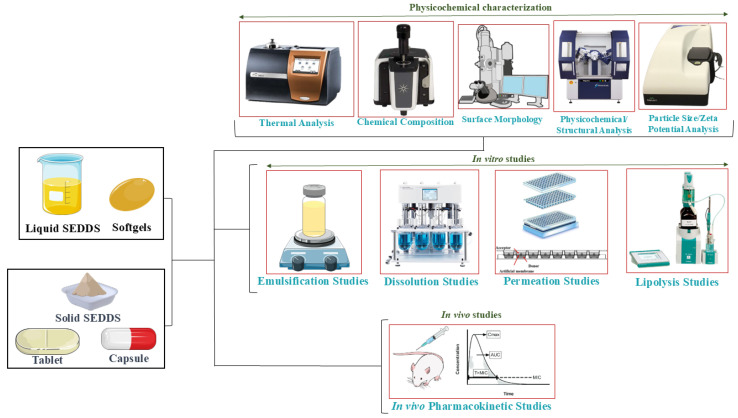
Key techniques for the characterization and evaluation of SEDDS formulations.

In addition to the methods used for liquid SEDDS, solid SEDDS (S-SEDDS) require evaluation of their solid state of a drug (PXRD and DSC), drug–excipient interactions (IR spectroscopy and DSC), and morphological characterization (SEM and TEM), as well as polymorphic transition (PXRD, Raman spectroscopy, and IR spectroscopy) [108,109]. These solid-state characterization methods complement traditional techniques and play a vital role in optimizing the performance of S-SEDDS. Furthermore, to enable the production of capsules or tablets and pass the prescribed limits for content uniformity and weight variation, SEDDS-loaded powder must have acceptable flow qualities. Powder flowability can be precisely measured by calculating the angle of repose and Hauser ratio and Carr’s index for evaluating powder compressibility, a crucial factor in tablet production [110,111]. Figure 4 represents the different techniques used for the characterization and evaluation of S-SEDDS. On top of that, various commonly used characterization tests are described in Table 7.

## 5. Applications of SEDDS

### 5.1. Enhanced Bioavailability in SEDDS

The key advantage of SEDDS is their ability to enhance the bioavailability of hydrophobic drugs by forming oil-in-water emulsions upon contact with gastric fluids. These emulsions have micro- or nano-sized droplets, significantly smaller than those in conventional emulsions, which increases the surface area for drug release and promotes faster, more efficient absorption. However, liquid SEDDS often require high concentrations of surfactants to solubilize the drug, posing a risk of toxicity, in addition to potential precipitation of the drug during storage, incompatibilities with gelatin capsules, and the high cost of production [118]. To address this, modifications such as solid SEDDS (S-SEDDS) and supersaturable SEDDS (Su-SEDDS) have been developed. Su-SEDDS solidify the liquid formulation using solid carriers, improving stability and handling while also maintaining the drug in a supersaturated state for extended periods. By incorporating polymeric precipitation inhibitors, Su-SEDDS prevent drug crystallization, further enhancing the bioavailability of poorly soluble drugs while reducing the need for excessive surfactants [119]. Recent applications to these modifications are outlined in Table 8.

### 5.2. Controlled-Release Formulations in SEDDS

Self-emulsifying drug delivery systems (SEDDS) are known for enhancing drug solubility and facilitating lymphatic absorption, bypassing first-pass metabolism. While these systems promote rapid drug absorption, they can lead to fluctuations in plasma concentrations, especially for drugs with narrow therapeutic indices or short half-lives. This poses challenges for managing conditions like diabetes, hypertension, or cardiovascular diseases, where maintaining stable drug levels is critical. To address these concerns, controlled release SEDDS formulations have been developed. These formulations balance enhancing bioavailability through lipid-based systems with providing a controlled release profile, ultimately improving therapeutic outcomes [125].

#### 5.2.1. Osmotic SEDDS Systems

Osmotic pumps within SEDDS formulations enable near-zero-order kinetics, ensuring a steady and controlled drug release over time. By employing osmotic agents such as mannitol or sodium chloride, water is drawn into the system through a semipermeable membrane, creating osmotic pressure that pushes the drug out through a precision-drilled orifice. The release kinetics are influenced by the orifice size and the coating materials, such as cellulose acetate or ethyl cellulose. For example, an osmotic SEDDS developed for Isradipine, a poorly soluble antihypertensive drug with a short half-life and poor bioavailability (about 24%), achieved controlled release for up to 12 h while enhancing bioavailability. The formulation incorporated mannitol, fructose, and citric acid as osmotic agents, and factors like orifice size and coating material were optimized to maintain a zero-order release profile [119,120].

#### 5.2.2. Floating and Gastroretentive SEDDS

For drugs with site-specific absorption windows in the gastrointestinal tract (e.g., stomach or intestines), controlled release can be enhanced through gastroretentive or enteric-coated systems that can target drug release to the desired location and ensure greater concentration at the absorption site and improved bioavailability. Similarly, floating drug delivery systems (FDDS) are particularly beneficial for hydrophobic drugs that are unstable in alkaline environments or have short half-lives. SEDDS increase the drug solubility by forming fine emulsions in the gastric environment, while FDDS prolong gastric retention by incorporating floating excipients. Combining SEDDS and FDDS enhances drug stability and absorption and extends the release profile [59,125]. Ginkgolides are promising candidates for this application due to their broad applications, including anti-thrombosis, anti-inflammatory, anti-ischemic, and neuroprotective effects. However, their clinical utility is hindered by challenges such as low solubility, inactivation in alkaline environments, and short half-lives, necessitating frequent dosing. Thus, solid SNEDDS formulations have been developed to enhance solubility and bioavailability and sustain drug supersaturation. Syloid^®^ XDP3050 and sodium bicarbonate further optimize the floating ability by reducing the tablet density and generating carbon dioxide, resulting in a zero-order release profile that maintains a stable drug release of 80% over 12 h, with minimal plasma concentration fluctuations [126]. Few case studies for osmotic SEDDS and floating/gastroretentive SEDDS are presented in Table 9.

#### 5.2.3. Mucoadhesive and SEDDS Combinations

Mucoadhesive polymers present an alternative effective strategy for extending drug release when combined with SEDDS. These polymers adhere to the intestinal mucosa, increasing the drug’s contact time at the absorption site and enhancing bioavailability. Thiolated polymers and surfactants, which form covalent disulfide bonds with cysteine residues in mucus glycoproteins, are particularly effective at strengthening adhesion and prolonging drug retention [127]. Chitosan, a mucoadhesive polymer, binds to mucin via electrostatic interactions due to its positively charged amino groups. Modifications like N-acylation can enhance chitosan’s lipophilicity, further promoting hydrophobic interactions with the mucus layer. A chemically modified acyl chitosan SNEDDS formulation for cefixime showed higher plasma drug concentrations compared to both traditional SNEDDS and pure drug controls [128]. Likewise, buccal patches with SEDDS fibers electrospun alongside thiolated polyacrylic acid fibers increased the buccal residence time by 200-fold, demonstrating improved penetration of lipophilic drugs like curcumin [129].

#### 5.2.4. Ionic Drug–Polymer Binding in SEDDS

This method of controlling drug release in SEDDS involves ionic interactions between hydrophilic drugs, such as the anionic drug captopril, and lipophilic cationic methacrylate copolymers (e.g., Eudragit RS, RL, and E), resulting in a more controlled release profile. Normally, hydrophilic drugs exhibit rapid release due to their high solubility in aqueous environments. However, by forming an ionic complex with lipophilic excipients, these drugs are retained longer within the oily droplets of SEDDS. This gradual release ensures extended therapeutic effects, which is particularly beneficial for drugs that are quickly eliminated from the body. In addition to improving the release profile, this system also protects captopril from enzymatic degradation in the gastrointestinal tract [130].

**Table 9:** Literature review on self-emulsifying drug delivery systems with controlled-release properties.

**Osmotic-SEDDS**				
**Drug**	**Challenge**	**Osmotic Excipients**	**Outcome**	**Ref.**
**Nifedipine**	Water insoluble and exhibit low bioavailability.	Mannitol and lactose	Improved solubility and cumulative release of about 84% in 12 h. Impact of orifice size, membrane composition and thickness on release profile was studied.	[131]
**Carvedilol**	Poor bioavailability of 20% due to low water solubility and first pass metabolism.	Mannitol	Cumulative release of about 85% in 12 h. Stable plasma drug concentration in comparison to marketed tablet. Relative bioavailability of 156.78% in beagle dogs.	[132]
**Vinpocetine**	Poor bioavailability of about 7% due to poor water solubility and first pass metabolism. Weak base with pH dependent solubility and short half-life.	Sodium chloride	Solubility was improved at higher pH values with extended drug release at a zero-order rate. Improved bioavailability in rabbits against marketed formulation. Impact of coating material, concentration of coating solution, and number of drills on drug release was studied.	[133]
**Nimodipine**	Poor water solubility and first pass metabolism.	Sodium chloride	Osmotic pump capsules were developed with zero-order release. The drug release was optimized based on key factors such as the amount of plasticizer, coating mass, and orifice size	[134]
**Floating/Gastroretentive Drug delivery systems**				
**Drug**	**Challenge**	**Floating/** **Gastroretention excipients**	**Outcome**	**Ref.**
**Fenofibrate**	Low solubility especially in alkaline conditions	Bilayer tablet composed of drug layer and swelling layer of HPMC	90% drug release attained in 12 h. Tablet swelled to a size that is bigger than the pylorus diameter	[135]
**Tetrahydrocurcumin**	Low aqueous solubility and short retention at the site of action (upper gastrointestinal tract)	Sodium bicarbonate and tartaric acid	Short floating lag time and extended floating capacity. 72% drug release in 12 h in addition to three-to-five-fold enhanced permeability via Caco-2 cell monolayers	[66]
**Tetrahydrocurcumin**	Low aqueous solubility and short retention at the site of action (upper gastrointestinal tract)	Different concentrations of glyceryl behenate to the floating pellets	80% drug release within 8 h in contrast to the liquid SEDDS that released the same amount within 2 h	[136]

### 5.3. Targeted Drug Delivery in SEDDS

SNEDDS can deliver drugs more efficiently to target sites by incorporating targeting ligands or specific molecules that direct the drug-loaded nano/microemulsions to particular tissues or cells. The targeting strategies can further reduce systemic side effects and enhance the overall therapeutic precision. This approach has been used to target Fisetin spheroids to the colon by mixing the SNEDDS with a mixture of guar gum, xanthan gum, and pectin. The colon-targeted system showed a 4.4-fold increase in bioavailability and a C_max_ of 7 h corresponding to the delayed release of the spheroids [137]. Similarly, Enoxaparin-coated SEDDS effectively targeted docetaxel (DTX) to drug-resistant tumors by optimizing DTX solubilization and partitioning. Enoxaparin played a crucial role by significantly enhancing the intracellular retention of DTX through the inhibition of key efflux transporters, such as MRP1 and BCRP. Additionally, Enoxaparin facilitated FGFR1-triggered endocytosis, boosting the uptake of docetaxel into cancer cells and further improving its therapeutic efficacy [138]. Hyaluronic acid was used for targeting ciprofloxacin SNEDDS against Salmonella typhi due to its ability to bind to CD44 and Toll-like receptor 4 (TLR4), which are overly expressed in intestinal infections. The effectiveness was evidenced by a four-fold permeability enhancement in goat intestine and 80% drug release over 72 h [139].

### 5.4. Recent Modifications in SEDDS

#### 5.4.1. SEDDS Combined with 3D Printing

The integration of SEDDS with 3D printing technology is rapidly evolving, offering the dual advantage of enhancing drug bioavailability and enabling precision medicine. Three-dimensional printing provides the flexibility to customize dosage forms, allowing for patient-specific formulations, while SEDDS improves the solubility and absorption of poorly water-soluble drugs. This combination opens up new possibilities for personalized therapeutics, especially for conditions requiring tight dose control or complex release profiles. For instance, researchers have developed 3D-printed capsules shells using FDM printing to enclose cyclosporin SNEDDS for the treatment of autoimmune diseases. These 3D-printed capsules offer not only enhanced bioavailability of cyclosporin but also the capability to tailor drug release and dosage based on individual patient needs [140]. Other examples include lidocaine suppositories [141], curcumin, and lansoprazole tablets using bioprinters [102] and dapagliflozin tablets using Biobot 1 (3D printer) [105].

#### 5.4.2. Self-Double Emulsifying Drug Delivery Systems

**Figure 5:**
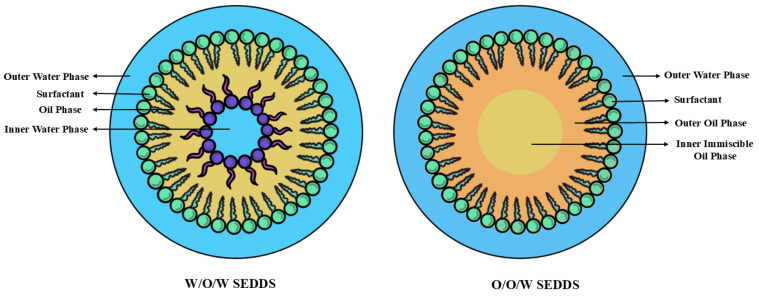
Schematic representation of a self-double-emulsifying drug delivery system.

This advanced approach is particularly effective for both hydrophilic and hydrophobic drugs. Upon dilution, SEDDS create fine double emulsions, such as water-in-oil-in-water (w/o/w) or oil-in-oil-in-water (o/o/w) systems, as presented in Figure 5. These double emulsions enable encapsulation of drugs in either the inner water or oil phase droplets, enhancing their bioavailability and stability. The w/o/w SDEDDS are highly effective for the delivery of BCS Class I and III drugs like hormones, enzymes, and certain anticancer and antiviral therapies. For instance, a novel self-double-nanoemulsifying system significantly enhanced the permeability and absorption of Zanamivir, an antiviral drug with poor oral absorption (up to 5%) due to low intestinal permeability [142]. Similarly, w/o/w SNEDDS of Imatinib mesylate, developed using aqueous phase titration, showed superior drug release and a 20% improvement in efficiency compared to the pure drug in treating colon cancer [143].

## 6. Challenges and Limitations of SEDDS

Self-emulsifying drug delivery systems (SEDDS) have emerged as a powerful strategy for enhancing the solubility and bioavailability of poorly water-soluble drugs, particularly those falling under the Biopharmaceutical Classification System (BCS) Classes II and IV. However, their widespread application is hindered by several challenges and limitations that need to be addressed. Stability issues are among the most significant hurdles, especially in liquid SEDDS [14]. These formulations are susceptible to phase separation, drug precipitation, and oxidative degradation during storage, particularly under fluctuating environmental conditions such as temperature and humidity [23]. Additionally, encapsulating liquid SEDDS in gelatin capsules often leads to leakage, further compromising their stability and shelf life. Such drawbacks underscore the necessity for advanced stabilization strategies, including the development of solid SEDDS, which offer enhanced physical and chemical stability but come with their own set of challenges [144].

The high surfactant concentrations required for efficient self-emulsification pose another significant limitation. While surfactants are essential for reducing interfacial tension and forming fine emulsions, their excessive use—often ranging from 30% to 60% of the formulation—can lead to gastrointestinal irritation and cytotoxic effects. This concern is particularly relevant for chronic therapies, where repeated exposure to high surfactant levels may affect patient compliance and safety. Furthermore, the selection of pharmaceutically acceptable, non-toxic surfactants with an optimal hydrophilic–lipophilic balance (HLB) remains a critical bottleneck in formulation design [3]. The issue is further compounded by the potential for drug precipitation upon dilution in gastrointestinal fluids. Supersaturation, which occurs when the solubilized drug exceeds its saturation concentration, can result in reduced therapeutic efficacy. Strategies such as incorporating precipitation inhibitors, polymeric stabilizers, or optimizing excipient ratios are actively being explored to address this issue [2].

The limited drug-loading capacity of SEDDS formulations also restricts their applicability. Most lipid-based systems can solubilize only small amounts of drugs, making them unsuitable for high-dose medications or drugs with extremely poor solubility in the lipid phase. Manufacturing scalability presents an additional challenge, as transitioning from lab-scale to industrial-scale production demands precise control over the excipient ratios, emulsification efficiency, and batch consistency. Techniques such as spray drying, hot-melt extrusion, and adsorption onto solid carriers, though promising for solid SEDDS, introduce additional complexity, cost, and potential compatibility issues [15]. Moreover, the lack of standardized regulatory guidelines and evaluation protocols further complicates the development process. This regulatory uncertainty makes it difficult for formulators to predict approval timelines and navigate the market introduction of novel SEDDS formulations [21].

Finally, while SEDDS have shown excellent potential for lipophilic drugs, their application for hydrophilic and macromolecular drugs remains limited [58]. Recent innovations, such as ionic liquids and hybrid delivery systems, offer promising solutions but require further exploration [145]. Despite these challenges, advances in computational modeling, innovative excipient engineering, and hybrid drug delivery approaches continue to push the boundaries of SEDDS formulations. Addressing these limitations through a multidisciplinary approach will be key to unlocking their full potential in clinical settings and expanding their role in modern pharmaceutical sciences.

## 7. Future Trends

Self-emulsifying drug delivery systems (SEDDS) are continuously evolving, driven by advancements in technology and innovative approaches to overcome existing limitations. The future of SEDDS lies in integrating cutting-edge techniques and expanding their applications across diverse therapeutic areas, particularly in addressing the challenges associated with stability, scalability, and targeted delivery.

### 7.1. Personalized Medicine

The paradigm shift toward personalized medicine has opened up new avenues for tailoring SEDDS to individual patient needs. The integration of 3D printing technology with SEDDS formulations has enabled the development of patient-specific dosage forms, offering precise control over drug release profiles and dose optimization. For instance, 3D-printed SEDDS-based systems can incorporate multiple drugs within a single unit, facilitating complex therapeutic regimens such as cancer combination therapies or tailored delivery for metabolic disorders. Recent studies have demonstrated the feasibility of creating bioactive self-nanoemulsifying drug delivery systems (SNEDDS) using 3D printing, highlighting their potential in producing multi-drug formulations with customized release kinetics. This approach ensures not only patient compliance but also enhanced therapeutic outcomes by addressing the inter-individual variability in drug metabolism and response [102,141].

### 7.2. In Silico Formulation Development

In silico techniques are revolutionizing the way SEDDS are designed and optimized, reducing reliance on labor-intensive experimental approaches. Computational methods, such as molecular docking and molecular dynamics (MD) simulations, enable the prediction of drug–excipient compatibility, solubility parameters, and emulsification efficiency. These methods help formulators select the most suitable combination of oils, surfactants, and co-surfactants to achieve optimal bioavailability. Moreover, machine learning algorithms are being employed to predict critical quality attributes like droplet size, zeta potential, and drug release profiles based on datasets of excipient properties. By integrating in silico modeling with experimental validation, researchers can significantly accelerate the formulation development process, improving both cost efficiency and formulation robustness [146].

### 7.3. Biologics and Peptide Delivery

Expanding the application of SEDDS to biologics and peptide drugs is a transformative trend. Biologics, such as monoclonal antibodies, and peptide-based therapeutics, face challenges such as poor oral bioavailability, enzymatic degradation, and low membrane permeability. SEDDS offer a promising platform for stabilizing these macromolecules and protecting them from harsh gastrointestinal conditions. For example, reverse micelle-containing SEDDS have shown significant potential in enhancing the stability and absorption of peptides. By encapsulating peptides within the lipophilic core, SEDDS can bypass enzymatic degradation and improve permeability across the intestinal epithelium. This innovation holds immense promise for advancing the oral delivery of biologics, addressing a critical unmet need in pharmaceutical sciences [147,148].

### 7.4. Hybrid Drug Delivery Systems

The combination of SEDDS with other advanced drug delivery technologies is an emerging trend aimed at enhancing stability, targeting, and therapeutic efficacy. Hybrid systems integrate SEDDS with nanoparticles, liposomes, or polymer-based carriers to create multi-functional drug delivery platforms. For instance, lipid–polymer hybrid nanoparticles incorporating SEDDS have demonstrated improved drug-loading and controlled-release capabilities. These systems are particularly beneficial for cancer therapies, as they enable precise targeting of tumor tissues while reducing systemic side effects. Additionally, hybrid systems allow for dual drug delivery, combining lipophilic and hydrophilic drugs within a single formulation, expanding the therapeutic potential of SEDDS [149].

### 7.5. Supersaturable SEDDS (Su-SEDDS)

Supersaturable SEDDS (S-SEDDS) represent a significant advancement in overcoming drug precipitation issues associated with conventional SEDDS. By incorporating precipitation inhibitors such as hydrophilic polymers, S-SEDDS maintain drugs in a supersaturated state upon dilution in gastrointestinal fluids, maximizing the absorption and bioavailability. Recent research has highlighted the success of S-SEDDS in delivering highly lipophilic drugs with enhanced pharmacokinetic profiles and reduced variability in drug release. These systems are particularly relevant for drugs with narrow therapeutic windows, where precise control over drug solubilization and release is critical [119].

### 7.6. Targeted and Responsive SEDDS

The development of stimuli-responsive SEDDS marks a new frontier in targeted drug delivery. By incorporating excipients that respond to specific physiological triggers such as pH, temperature, or enzymatic activity, these systems enable site-specific drug release. For example, pH-responsive SEDDS can release drugs selectively in the acidic environment of the stomach or the neutral pH of the intestines, enhancing the therapeutic efficacy while minimizing off-target effects. This approach is particularly promising for oncology, where localized drug delivery to tumor tissues can significantly improve outcomes. Additionally, ligand-based targeting using moieties such as hyaluronic acid or folic acid enhances the specificity of SEDDS for receptors overexpressed on diseased cells, further expanding their potential applications [138,150].

**Figure 6:**
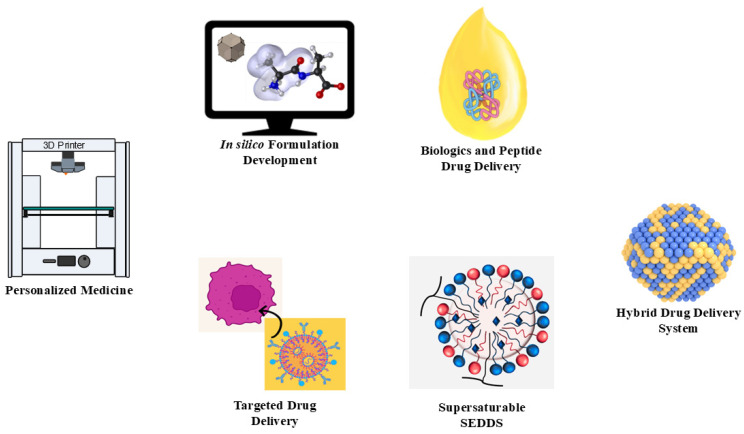
Emerging trends in SEDDS innovation: exploring personalized medicine, in silico design, biologics delivery, hybrid systems, supersaturable SEDDS, and targeted-responsive formulations.

The future of SEDDS lies in its ability to integrate advanced technologies and address existing challenges to expand its therapeutic scope. From personalized medicine enabled by 3D printing to the incorporation of biologics and hybrid delivery systems, SEDDS are poised to play a transformative role in drug delivery. Innovations such as S-SEDDS, in silico modeling, and stimuli-responsive formulations promise to overcome the current limitations, offering enhanced stability, bioavailability, and patient compliance. These advancements, as presented in Figure 6. will not only unlock new therapeutic possibilities but also establish SEDDS as a cornerstone of modern pharmaceutical science.

## 8. Conclusions and Perspectives

Self-emulsifying drug delivery systems (SEDDS) have revolutionized the field of drug delivery by addressing the critical challenges of solubility and bioavailability in poorly water-soluble drugs. This review has comprehensively covered the multifaceted aspects of SEDDS, including their fundamental principles, formulation strategies, characterization techniques, and wide-ranging therapeutic applications. The classification of SEDDS into conventional SEDDS, self-microemulsifying drug delivery systems (SMEDDS), and self-nanoemulsifying drug delivery systems (SNEDDS) underscores the diversity of these systems in achieving specific therapeutic objectives. Each category is tailored to meet unique demands, with differences in droplet size, stability, and bioavailability enhancement.

The transition from liquid to solid SEDDS has further expanded the versatility of this delivery system. Solid SEDDS, developed through techniques such as spray drying, adsorption onto carriers, and hot-melt extrusion, address the stability, portability, and manufacturing challenges inherent to liquid SEDDS. This transition has allowed for better scalability, enhanced shelf-life, and improved patient compliance while still retaining the advantages of self-emulsifying systems. Characterization remains a cornerstone of SEDDS development, providing critical insights into their performance and stability. Methods such as droplet size analysis, cloud point determination, and in vitro dissolution studies for liquid SEDDS, coupled with solid-state characterization techniques like SEM, DSC, and XRPD for solid SEDDS, ensure these systems meet stringent quality standards. These evaluations bridge the gap between innovative formulation design and clinical application, providing a robust foundation for further optimization.

The applications of SEDDS extend far beyond bioavailability enhancement. From enabling controlled and targeted drug release to stabilizing and delivering biologics and peptides, these systems have demonstrated their adaptability to diverse pharmaceutical challenges. Emerging innovations, such as hybrid drug delivery systems, 3D-printed SEDDS, and supersaturable SEDDS (Su-SEDDS), are redefining the boundaries of this technology, ensuring greater therapeutic impact and patient-centric solutions. While SEDDS hold significant promise, they also face various challenges. Issues such as excipient toxicity, drug precipitation, and scalability persist, emphasizing the need for continued innovation and collaboration. Computational modeling and in silico approaches are already playing a vital role in overcoming these challenges by accelerating formulation development and ensuring precision in excipient selection.

In conclusion, SEDDS represent a paradigm shift in pharmaceutical development, combining scientific innovation with practical applicability. By addressing unmet needs and leveraging emerging technologies, SEDDS are poised to remain a cornerstone in the development of next-generation drug delivery systems, shaping the future of modern healthcare.

## Figures and Tables

**Table 1:** Commercially available SEDDS-based formulations and their therapeutic applications.

Product Name	Drug	Therapeutic Use	BCS Class	Marketed Dosage Form	Company
**Gengraf** ^**®**^	Cyclosporine A/III	Immunosuppressant	IV	Hard gelatin capsule	AbbVie Inc.
**Sandimmune** ^**®**^	Cyclosporine A/I	Prophylaxis against organ rejection in kidney, liver, and heart transplants	IV	Soft gelatin capsule	Novartis pharmaceuticals corporation
**Neoral** ^**®**^	Cyclosporine	Immunosuppressant	IV	Soft gelatin capsule	Novartis Pharmaceuticals Corporation
**Norvir** ^**®**^	Ritonavir	Adjunctive therapy with other antiretroviral agents during HIV-1 infection	II	Soft gelatin capsule	AbbVie Inc.
**Fortovase** ^**®**^	Saquinavir	HIV infection	IV	Soft gelatin capsule	Roche Laboratories Inc.
**Agenerase** ^**®**^	Amprenavir	HIV infection	II	Soft gelatin capsule	GlaxoSmithKline
**Aptivus** ^**®**^	Tipranavir	HIV infection	II	Soft gelatin capsule	Boehringer Ingelheim Pharmaceuticals, Inc.
**Depakene** ^**®**^	Valproic acid	Monotherapy and adjuvant therapy in during treatment of patients with complex partial seizures	II	Soft gelatin capsule	AbbVie Inc.
**Rocaltrol** ^**®**^	Calcitriol	Secondary hyperparathyroidism and hypocalcemia	II	Soft gelatin capsule	Roche Products Limited
**Targretin** ^**®**^	Bexarotene	Treatment of cutaneous manifestations of cutaneous T-cell lymphoma	II	Soft gelatin capsule	Ligand Pharmaceuticals/Eisai Ltd.
**Vesanoid** ^**®**^	Tretinoin	Induction of maturation of acute promyelocytic leukemia (APL)	II	Soft gelatin capsule	Roche Laboratories Inc.
**Accutane** ^**®**^	Isotretinoin	Severe recalcitrant nodular acne	II	Soft gelatin capsule	Roche Laboratories Inc.

**Table 2:** Components of recently investigated SEDDS formulations.

Oils	Surfactants	Co-Surfactants	Drugs	References
Capryol^®^ 90 (Propylene glycol monocaprylate)	Cremophor^®^ RH 40	PEG 400	Finasteride	[39]
Isopropyl palmitate (IPP) (Isopropyl ester of myristic acid)	Cremophor^®^ RH 40	PEG 200	Cepharanthine	[40]
Liquid paraffin	Tween 80	Propylene glycol	Metformin	[41]
Castor oil	Labrasol^®^	Transcutol^®^ (diethylene glycol monoethyl Ether)	Curcumin	[42]
Gelucire ^®^ 44/14	Gelucire ^®^ 48/16	Transcutol^®^	Ibuprofen	[43]
Capmul ^®^ MCM (Glyceryl monocaprylate)	Gelucire ^®^ 48/16	Propylene glycol	Quetiapine fumarate	[17]
Peceol^®^ (Glyceryl monooleate)	Labrasol^®^	Cremophor^®^ EL	Apixaban	[44]
Castor Oil, Labrafac^®^ (Medium-chain triglycerides)	Docusate sodium	Propylene glycol	Semaglutide	[45]
Isopropyl myristate	Labrafil^®^ (Polyoxyglycerides of linoleic acid./oleic acid)	PEG 400	Dasatinib	[46]
Labrafac^®^ lipophile	Kolliphor^®^ RH 40	Transcutol^®^	Carvedilol	[47]

**Table 3:** Comparison of SEDDS, SMEDDS, and SNEDDS [30,53,54].

Characteristics	SEDDS	SMEDDS	SNEDDS
**Mean droplet size**	250 nm–5 µm	100–250 nm	<100 nm
**Appearance**	Turbid/Cloudy	Clear to translucent	Optically clear
**Solubilizing capacity**	High	High	High
**Stability**	Thermodynamically unstable	Thermodynamically stable	Kinetically stable
**Bioavailability**	Moderate	Enhanced	Superior
**Oil Types**	Long-chai–n triglycerides (e.g., soybean oil, olive oil)	Medium-chain triglycerides (e.g., Labrafac^®^, Captex^®^ 355) (MC triglycerides)	Medium- and short-chain triglycerides (e.g., Capmul^®^, Miglyol^®^) (caprylic and capric triglycerides)
**HLB of surfactants**	<10	10–12	>12
**Co-surfactants**	Not essential	Short-chain alcohols (e.g., propylene glycol)	Polyethylene glycol (PEG), Transcutol^®^

**Table 4:** Comparison Lipid Formulation Classification System (LFCS): Composition, Characteristics, Applications, and SEDDS Types [8,30,51].

Type	Composition	Behavior in Aqueous Media	Characteristics	Applications	SEDDS Type
**Type I**	Pure oils (long- or medium-chain triglycerides)	Requires bile salts for emulsification	Simple, digestion-dependent	Nutraceuticals, lipid-soluble vitamins	Not applicable
**Type II**	Oils + lipophilic surfactants	Spontaneously emulsifies in GI fluids	Self-emulsifying, digestion-independent	SEDDS for poorly soluble lipophilic drugs	Conventional SEDDS
**Type IIIa**	Oils + high-HLB surfactants (<50% surfactant)	Forms microemulsions with bile salts	Improved bioavailability, partially digestion-dependent	SMEDDS for intermediate solubility drugs	SMEDDS
**Type IIIb**	Oils + high-HLB surfactants (>50% surfactant)	Forms nanoemulsions with bile salts	Higher surfactant content, enhanced emulsification	SNEDDS for poorly soluble and permeable drugs	SNEDDS
**Type IV**	High-HLB surfactants and co-surfactants (no oil)	Forms colloidal micelles	Lipid-free, relies on surfactant solubilization	Hydrophilic drug delivery	Not applicable

**Table 5:** Comparative overview of liquid and solid SEDDS.

Aspect	Liquid SEDDS	Solid SEDDS
**Advantages/Disadvantages**		
**Stability**	Moderate; prone to stability issues like phase separation over time, affecting product quality and dosing accuracy [62].	More stable; solid state can improve storage stability and enhance dosing consistency [18].
**Manufacturing Complexity**	The scalability is challenging in L-SEDDs due to precision required in liquid encapsulation and potential drug precipitation issues, especially at lower temperatures [14].	More complex; may require additional solidification steps (e.g., spray drying, freeze drying) [14].
**Patient Compliance**	Easier to swallow (liquid or soft gel forms); more acceptable for pediatric/geriatric patients.	Bitter taste is a common challenge with many APIs, necessitating the use of excipients in SEDDS that provide a pleasant sensory experience to enhance patient compliance, while natural oils such as olive and corn oil are favorable for their taste, semi-synthetic, and synthetic excipients may contribute to bitterness [21].
**Bioavailability Enhancement**	Enhances bioavailability significantly by eliminating the dissolution step and promoting rapid drug absorption due to faster dispersion [39].	There is no significant difference between L-SNEDDS and S-SNEDDS in terms of bioavailability; however, S-SNEDDS exhibits slightly slower dispersion compared to L-SNEDDS due to additional steps such as disintegration and desorption [39].
**Performance in Drug Delivery**		
**Release Profiles**	Rapid release; beneficial for drugs needing quick onset of action [39].	Controlled release: suitable for sustained release applications but may require additional excipients to enhance emulsification [39].
**Absorption**	High initial absorption; effective for lipophilic drugs with low solubility [63].	May show delayed but prolonged absorption due to controlled release characteristics [63].
**Case Studies**	Often used in formulations where fast drug action is required, such as NSAIDs (e.g., ibuprofen) and cardiovascular drugs (e.g., nifedipine), to maximize their quick onset of action [64,65]	Utilized for drugs needing sustained or controlled release, like certain hormones (e.g., estradiol), anti-inflammatory drugs, and medications with a narrow therapeutic window, ensuring steady therapeutic levels [65].

**Table 7:** Overview of the characterization techniques for liquid and solid SEDDS: methods and significance.

Tests	Techniques	Significance	Description	References
**Self-emulsification test**	Visually	When in contact with water while being moderately stirred, an ideal SEDDS formulation has the capacity to spontaneously form an emulsion. The presence of a clear, isotropic, transparent solution denotes the development of a microemulsion, whereas an opaque, milky white presence denotes the formation of a macroemulsion. The formulation is thought to be stable if there is no precipitation and/or no phase separation.	Self-emulsification assessment is aided by visual observation to evaluate the emulsification behavior of SEDDS.	[17,107]
**Mean droplet size assessment, Zeta Potential**	DLS (Dynamic light scattering)	The size of the droplet is influenced by the type and concentration of the surfactant. The microemulsion formed when SEDDS is dissolved in water has a very narrow droplet size distribution, which is vital for efficient drug release, in vivo absorption, and stability. The zeta potential represents the stability of the emulsion after dilution. The formulation remains stable if the zeta potential is greater. Particles with a zwitterion charge have superior biocompatibility and a longer blood residence duration than particles with any surface charge.	DLS methods are used to analyze droplet size and zeta potential.	[70]
**Solid state characterization**	DSC (Differential Scanning Calorimetry), PXRD(Powder X-Ray Diffraction)	DSC is a thermal analysis technique in which SEDDS formulations are put through linear heating and cooling cycles in order to learn about their melting, glass transition, decomposition and recrystallization. The diffraction pattern of crystalline material is measured by powder X-ray diffraction (PXRD). Phase identification, sample purity, crystallite size, and, in some situations, morphology is just a few of the crucial details that powder XRD analysis of a sample can provide in addition to numerous microscopic and spectroscopic techniques.	DSC investigations characterize the physical state of S-SEDDS. Using DSC, quantitative behavior of thermotropic phase transitions can be determined. Melting peaks can be observed in the thermograms after analysis. Crystallinity and amorphous content of SDEDS can be determined using powder X-ray diffraction (PXRD).	[112,113]
**Morphological characterization**	Scanning electron microscopy (SEM), Transmission electron microscopy (TEM)	SEM, or scanning electron microscopy, is an imaging method commonly employed to determine the shape, composition, and size of solid samples. An electron beam is focused on the surface of the sample to create an image of its structure. TEM is also a widely used technique determine the size, shape and surface topography of samples.	The surface characteristics of active pharmaceutical ingredients and excipients utilized in various SEDDS formulations are investigated using this technique.	[114]
**In vitro release studies**	Dissolution apparatus	In pharmaceutical drug development, the in vitro dissolution test is commonly used to ascertain batch production uniformity by gathering data on drug release for quality control. Also, this test can be used to foretell how the drug formulation would behave in vivo, and later to determine the in vivo–in vitro relationship between drug absorption and its release from the formulations.	Dissolution testing is useful for selecting the best formulation among all those prepared using different excipients, with the goal of selecting the optimal formulation that displays the most desirable and reproducible dissolution profile.	[115]
**In vitro lipolysis**	pH-stat lipolysis model	The SEDDS contain lipidic components that are susceptible to GIT digestion, which may have a positive or negative on the solubilization of drugs. Gastric lipase- and intestine-based enzymes break down lipids in the stomach, producing amphiphilic digestion products that self-assemble to form liquid crystalline formations at droplet interfaces. As these structures are dispersed, various phases are produced, and these phases have various drug-solubilizing capacities. This test is used to examine drug solubilizing capacity and potential interactions with biliary components.	A temperature-controlled reaction vessel with digesting medium is used to simulate intestinal and gastric environment.	[116,117]

**Table 8:** Case studies on recently developed SEDDS demonstrating enhanced bioavailability.

Drug	Lipid/ Surfactant/ Co-surfactant	Solid/ Precipitation InhibitoR	Particle Size of SEDDS	Outcome	Ref.
**Thymoquinone**	Olive oil, Tween 80, Span 85 (Sorbitan trioleate) and PEG 300	N/A	158 nm	Enhanced absorption and bioavailability. Reduced oxidative stress and improved cell survival in rats from ischemia/reperfusion (I/R) injury	[120]
**Quetiapine fumarate**	Capmul MCM (MC mono-, di-, and triglycerides, MC mixed glycerides), Gelucire 48/16, and propylene glycol	Klucel EF	92 nm	Extended-release profile for 24 h and stable formulation for 3 months under accelerated conditions	[17]
**Quercetine**	Capmul MCM EP, Tween 20 and ethanol	HPMC E5	127–270 nm	1.25fold higher drug content in aqueous environment, 2.2fold, and 2fold increase in C_max_ and AUC in comparison to SEDDS without precipitation inhibitor	[121]
**Sorafenib**	Peceol, Labrasol and Transcutol HP	HPMC and PVP	334–430 nm	Improvement in dissolution rate, cel uptake, and pharmacokinetic parameters.	[122]
**Cilostazol**	Oleic acid, tween 80, propylene glycol, Transcutol	Avicel 200, Aerosil 101	154–425 nm	High dissolution profile under sink and non-sink condition in comparison to marketed formulation	[123]
**Valsartan**	Capryol90 (Propylene glycol monocaprylate-type II), Transcutol HP, and Tween 20	Avicel pH 101	67.5 and 177 nm	Enhanced permeability for liquid and solid SEDDS against commercial product	[124]
**Curcumin**	Castor oil, Lexol, Labrasol, Cremophor RH 40 and Transcutol	Neusilin UFL2	About 100, and 150 nm	Enhanced dissolution profile, permeation and cellular uptake	[42]

## Abbreviations

SEDDS	Self-emulsifying drug delivery systems
SMEDDS	Self-microemulsifying drug delivery systems
SNEDDS	Self-nanoemulsifying drug delivery systems
L-SEDDS	Liquid self-emulsifying drug delivery systems
S-SEDDS	Solid self-emulsifying drug delivery systems
Su-SEDDS	Supersaturable self-emulsifying drug delivery systems
LFCS	Lipid Formulation Classification System
W/O/W	water-in-oil-in-water
PVA	PolyvinylAlcohol
(HP-β CD)	Hydroxypropyl-β Cyclodextrin
HPMCAS	Hydroxypropyl Methylcellulose Acetate Succinate
MCC	MicrocrystallineCellulose
DSC	Differential Scanning Calorimetry
TEM	Transmission electron microscopy
DLS	Dynamic Light Scattering
DTX	Docetaxel
BCS	Biopharmaceutics Classification System
HLB	Hydrophilic–Lipophilic Balance
PEG	Polyethylene Glycol
GIT	Gastrointestinal tract
UWL	Unstirred water layer
LPs	Lipoproteins
O/W	oil-in-water
O/O/W	oil-in-oil-in-water
Na-CMC	Sodium Carboxymethyl Cellulose
PEO	Polyethylene Oxide
HPC	Hydroxypropyl Cellulose
PXRD	Powder X-ray diffraction
SEM	Scanning electron microscopy
IR	Infra-red
FDDS	Floating drug delivery systems
MD	Molecular dynamics
